# Gene silencing by RNAi in mouse Sertoli cells

**DOI:** 10.1186/1477-7827-6-29

**Published:** 2008-07-11

**Authors:** Emilio González-González, Pedro P López-Casas, Jesús del Mazo

**Affiliations:** 1 Department of Cell and Developmental Biology, Centro de Investigaciones Biológicas, CSIC, Ramiro de Maeztu 9, 28040 Madrid, Spain

## Abstract

**Background:**

RNA interference (RNAi) is a valuable tool in the investigation of gene function. The purpose of this study was to examine the availability, target cell types and efficiency of RNAi in the mouse seminiferous epithelium.

**Methods:**

The experimental model was based on transgenic mice expressing EGFP (enhanced green fluorescent protein). RNAi was induced by in vivo transfection of plasmid vectors encoding for short hairpin RNAs (shRNAs) targeting EGFP. shRNAs were transfected in vivo by microinjection into the seminiferous tubules via the rete testis followed by square wave electroporation. As a transfection reporter, expression of red fluorescent protein (HcRed 1) was used. Cell types, the efficiency of both transfections and RNAi were all evaluated.

**Results:**

Sertoli cells were the main transfected cells. A reduction of about 40% in the level of EGFP protein was detected in cells successfully transfected both in vivo and in vitro. However, the efficiency of in vivo transfection was low.

**Conclusion:**

In adult seminiferous epithelial cells, in vivo post-transcriptional gene silencing mediated by RNAi via shRNA is efficient in Sertoli cells. Similar levels of RNAi were detected both in vivo and in vitro. This also indicates that Sertoli cells have the necessary silencing machinery to repress the expression of endogenous genes via RNAi.

## Background

RNA interference (RNAi) describes any process in which double stranded RNA (dsRNA) triggers post-transcriptional gene silencing. Strategies for inducing gene silencing, either for the study of gene function or in a therapeutic context, have been developed [1]. Small interference RNAs (siRNAs) and short hairpin RNAs (shRNAs) have been used *in vitro *and *in vivo *for interfering with RNA [2-5]. siRNAs are dsRNAs of 21–23 base pairs (bp) generated by chemical synthesis [6], enzymatic cleavage [7] or expression systems [8], while shRNAs are dsRNA molecules that mimic endogenous pre-micro RNAs (pre-miRNAs). shRNAs consist of two palindromic sequences of 19–29 nucleotides (nt) with a short loop of single-stranded RNA (4–10 nt) at one end [9]. The RNAse III family of nucleases known as 'Dicer' binds and cleaves both pre-miRNAs and shRNAs into their mature 21–25 bp forms [9-12]. One strand of these miRNAs or siRNAs is incorporated into the RNA-induced silencing complex (RISC), which then either identifies, binds and cleaves the complementary messenger RNA [13,14] or induces translational repression [15]. Recent work indicates that shRNAs are more potent inducers of RNAi than siRNAs [16].

Silencing of specific mRNAs by RNAi has been used *in vivo *in the eye [17,18], brain [19-22], lung [23-26], skeletal muscle [27-30], liver, kidney, spleen [31-39] skin [40], and pancreas [41]. In the testis, the seminiferous epithelium of adults is organized into a complex structure composed of the germ cells and Sertoli cells. Sertoli cells, a somatic cell type, extend from the basement membrane of the seminiferous tubules to reach the lumen. The architectural pattern of these cells provides a structural framework for Sertoli cell-Sertoli cell and Sertoli cell-germ cell interactions. These interactions are based on intimate contacts through different types of junctions (e.g. occluding junctions, anchoring junctions and communicating junctions), supporting a specific microenvironment required by developing germ cells [42-45].

To transplant spermatogonial stem cells into the seminiferous epithelium, Brinster and Avarbock [46] developed an *in vivo *technique involving microinjection into the lumen [47]. Following this microinjection, Shoji et al. [48] introduced shRNAs expression vectors into the seminiferous tubules reporting gene silencing in the spermatogenic cells of prepubertal mice. However, in animals in which all seminiferous epithelium architectural structures are fully established, RNAi has yet to be studied.

This work reports the use of a transgenic mouse model expressing EGFP to determine which cells of the seminiferous epithelium are preferentially transfected by shRNA-coding plasmids for the induction of gene silencing and its efficiency. *In vitro *experiments were also performed to verify the efficiency of RNAi in Sertoli cells, the main transfected target cell seen in *in vivo *transfections.

## Methods

### Experimental animals

All the mice (*Mus musculus*) used in these experiments were bred at the Animal Care Facility of the Centro de Investigaciones Biológicas (CIB-CSIC) on a 12L:12D cycle. Male mice of the C57BL/6J wild type were used to investigate *in vivo *transfection efficiency. The C57BL/6 TgN(act-EGFP)OsbC14-Y01-FM131 (FM131) [49] transgenic mouse line, which constitutively expresses EGFP, was provided by RIKEN BRC (Japan). All procedures were performed according to the guidelines of the CSIC Bioethics Committee.

### Plasmids

Plasmid pEGFP-N1 (Clontech, Palo Alto, CA, USA), expressing EGFP as a reporter, was used as an *in vivo *transfection control. Plasmid pGtoR (a kind gift of Dr. Masaru Okabe, University of Osaka, Japan) was used to induce RNAi in EGFP. pGtoR contains the RNA polimerase III promoter H1 driving the expression of an shRNA containing 21 nt sense and antisense sequences homologous to an EGFP encoding region (shRNA-EGFP), as well as the CAG promoter controlling the expression of HcRed1 protein [50].

A vector called pRed, used as a negative control, was generated by digestion of pGtoR with BamH1 and HindIII followed by religation to eliminate the H1-shRNA-EGFP cassette. Consequently, pRed only expresses the HcRed1 protein.

### *In vivo *electroporation

**Figure 1:**
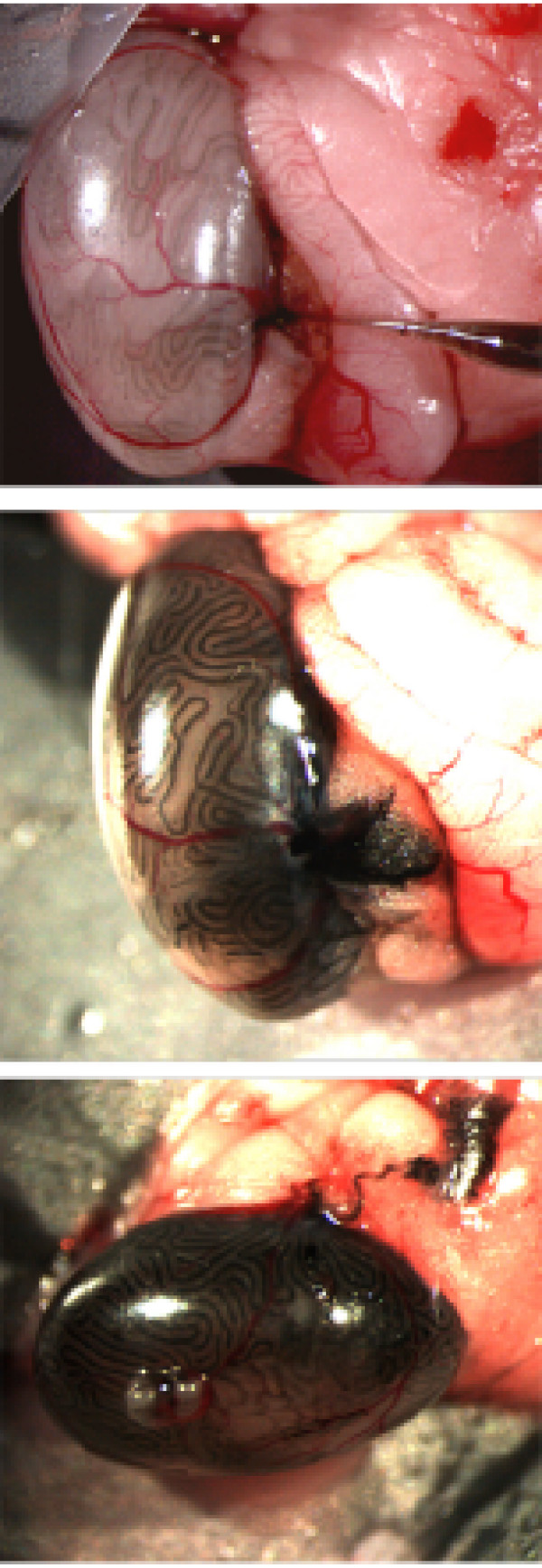
**Different phases of *in vivo *microinjection of vectors into testis tubules through the *rete testis***. Nigrosin was used as tracer.

Male mice of 30–45 days post-natal (dpn) were anaesthetized by an intraperitoneal injection of Rompun (Bayer, Kiel Germany)/Ketolar (Pfizer, Dublin Ireland) solution (315 μl/Kg; 84 mg/Kg respectively). After opening the abdominal cavity, the testes were exposed under a binocular microscope as previously described [47]. Approximately 20 μl of plasmid DNA in TE buffer (10 mM Tris, and 1 mM EDTA, pH adjusted to 7.5) (3 μg/μl) containing nigrosine (1 mg/ml) as a tracer was slowly microinjected into the rete testis using a 40–70 μm in diameter glass micropipette (Fig 1). Trypan blue, the standard tracer for procedures of this kind, was ruled out due to its autofluorescence. For *in vivo *electroporation, each testis was held between tweezer-type electrodes (model 520, 7 mm diameter, BTX, San Diego, CA) briefly soaked in PBS, and two sets of four electric pulses of square wave were applied (using an electric pulse generator ECM 830 [BTX]). Each pulse provided 50 V for 50 ms; the interval between the pulses was 950 ms [51]. The testes were then returned to the abdominal cavity and the skin stitched closed. Four days later the mice were sacrificed and the testes removed for analysis.

### Cytological examination

The testes were fixed with 4% paraformaldehyde in PBS, and passed through a series of 10, 20 and 30% sucrose. The samples were then placed in Tissue-Tek OCT (Sakura Finetek, The Netherlands) and frozen on dry ice. Cryosections (10 μm thick) were processed for histological examination by fluorescent microscopy using an inverted microscope (Nikon ECLIPSE TE300) (Tokio, Japan). In each experimental condition 10 mice were examined and 50 to 100 sections of the whole testis were assessed per mouse. A histopathological evaluation of the testis sections was performed on each specimen. Cultured Sertoli cells were also analysed by fluorescence microscopy. In both cases, Hoechst 33258 was used to counterstain the cell nuclei with 5 min incubation of the dye in PBS at 15 μg/ml.

### Isolation of primary Sertoli cells and their culture

Sertoli cells were isolated from FM131 mice as previously described [52] with minor modifications. As mature Sertoli cells can not be efficiently cultured, testes from 17 days post-natal (dpn) males were decapsulated in PBS, cut into small fragments and digested in DMEM:Ham's F12 medium (1:1, Gibco BRL, Eggenstein, Germany) containing 2% foetal bovine serum (FBS) (ICN Biomedical, Costa Mesa, CA, USA), 0.2 mg/ml collagenase-dispase (Roche, Mannheim, Germany) and 0.1 mg/ml DNAse I (Roche) for 30 min at 32°C. The resultant seminiferous tubule fragments were washed with DMEM:F12 followed by two additional digestions under the same conditions, and then washed again with DMEM:F12. This material was repeatedly passed through an 18 1/2 G needle and the disaggregated cells were collected by filtration through a 70 μm Cell Strainer (BD Falcon, Lexington, TN, USA). The cells were incubated with continuous shaking in DMEM:F12 containing 2% FBS, 0.4 mg/ml hyaluronidase I-S (Sigma St Louis, MO, USA) and 0.1 mg/ml DNAse I for 30 min at 32°C. The sample was then centrifuged at 200 g for 10 min. The Sertoli cells obtained were resuspended in DMEM:F12 with 10% FBS and allowed to settle (20 min at 32°C). The settled cells were cultured at 32°C in a 5% CO2 atmosphere for three days in DMEM:F12 supplemented with 10% FBS, 100 U/ml penicillin, 100 μg/ml streptomycin and 1× insulin-transferrin-sodium selenite media supplement (ITSS) (Sigma). The germ cells that had residually attached to the Sertoli cells were removed by hypotonic treatment with 20 mM Tris-HCl, pH 7.4 at 20°C for 3 min, and were cultured in supplemented DMEM:F12 medium.

To discern the presence of potential contaminant cells, analysis of transferrin (*Trf*) expression, as a Sertoli cell marker, was carried out by RT-PCR. As we previously reported to detect other potential contaminant cells RT-PCR analysis was performed for the expression of *Hsd17 *(17beta-hydroxysteroid dehydrogenase) as a Leydig cell marker and *S16 *(ribosomal protein) were also assessed as negative and positive controls respectively [53].

### *In vitro *transfection

Sertoli cells growing *in vitro *in wells were transfected with the different plasmids using 0.4 μg of plasmid per well (1.9 cm^2^) by FUGENE™6 reagent (Roche) according to the manufacturer's instructions. The cells were harvested three, five and seven days post-transfection. Each experiment was performed three times.

### Flow cytometry analysis

Monocellular suspensions of the testis cells were obtained from *in vivo *electroporated testes and controls by digestion of the tubules following a procedure similar to that performed for the isolation of the Sertoli cells. After hyaluronidase digestion, the monodispersed cells from the seminiferous epithelium were resuspended in PBS. Sertoli cells from *in vitro *cultures and monocellular suspensions of seminiferous tubule cells were then analysed in a Becton-Dickinson FACS Vantage flow cytometer (Mountain View, CA, USA). The average number of cells analysed per flow cytometry run in each experiment was 3 × 10^4 ^cells. Each experimental condition was repeated at least three times. Transfected cells were detected by the presence of HcRed1 excited at 630 nm and emission recorded at 660 nm. Green fluorescence intensities were measured in transfected cells by excitation at 488 nm and emission recorded at 530 nm and compared to both pGtoR and pRed transfected populations. Each value represents the mean of three individual experiments. Statistical analysis was performed using the Student t test for independent data. The significance was set at p < 0.05.

## Results and Discussion

### *In vivo *transfection

**Figure 2:**
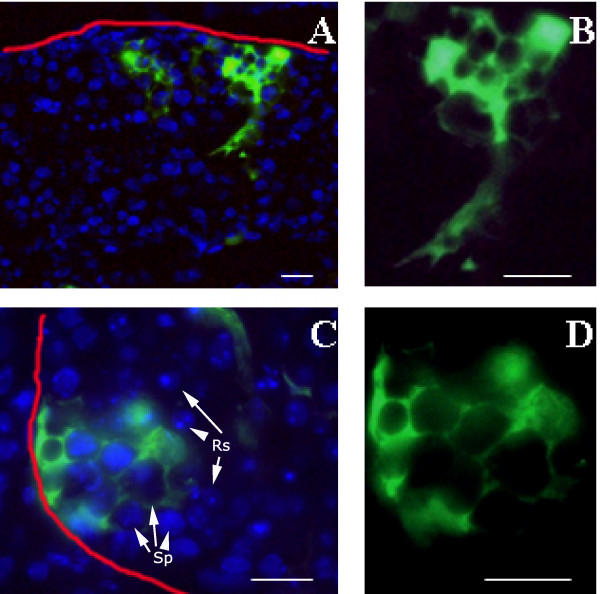
**Seminiferous tubules of testis from C57BL/6J wild type mice after *in vivo *transfection with the pEGFP-N1 vector**. **A **and **C**) Merge image of partial view of tubule sections showing EGFP-positive Sertoli cells (with the well-known arborescent-like cytoplasm) as preferentially transfected cells. Nuclei were stained with Hoechst 33258 dye. Spermatocytes (Sp) round spermatid (Rd) are indicated (**C**). The red lines indicate the basement membranes of seminiferous tubules. **B **and **D **show enlarged images of EGFP fluorescence taken 24 hours after transfection. Bars represent 50 μm.

To evaluate the efficiency of *in vivo *gene silencing in mouse testis, after completing the first wave of spermatogenesis, we first characterized the efficiency to deliver plasmid DNA into the cells of the seminiferous epithelium. To determine which cell types were preferentially transfected either pEGFP-N1 or pGtoR was used. The cytological detection of green or red fluorescent proteins indicated that Sertoli cells (Fig. 2) were the cell type most commonly transfected (less than 1% of germ cells were also transfected). However, the efficiency of transfection of Sertoli cells was always less than 10% although no differences were found between the plasmids used. As previously described, altering the experimental conditions, i.e., increasing voltage and/or the number of electrical pulses, was found to damage the seminiferous epithelium as assessed by histopathological analysis (data not shown) [51].

### *In vivo *gene silencing of seminiferous epithelial cells

Since shRNA molecules can induce potent gene silencing [9,16,54-56], vectors expressing shRNA were used in the present work to confirm the efficiency of silencing of a specific gene both *in vivo *in seminiferous tubule cells and in *in vitro *cultures of Sertoli cells.

**Figure 3:**
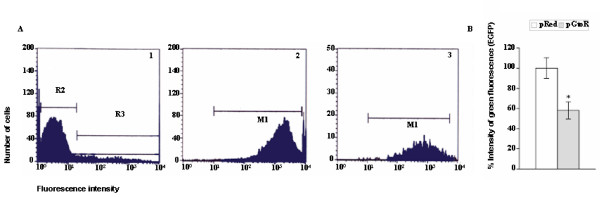
**Comparative results from flow cytometry analysis of EGFP-expression in seminiferous epithelium cells transfected *in vivo *with pRed or pGtoR**. **(A) **Representative traces of flow cytometry using pGtoR vector. 1) R2 corresponds to non-transfected cell population; R3 represent the transfected cells (red fluorescent). 2) M1 represents the level of green fluorescent (EGFP) of R2 cell population 3) represents the level of EGFP in transfected cells (R3). **B) **The histogram shows the mean (Mean ± SE) intensity of green fluorescence of the cells (EGFP) transfected *in vivo *with pRed or pGtoR, as determined by flow cytometry four days after transfections. Asterisk shows statistically significant differences as measured by p < 0.05.

The vector pHcRed1-shRNA-EGFP (pGtoR) [50] allows detection of transfected cells expressing shRNA-EGFP, based on the co-expression of the red fluorescence protein HcRed1. Nevertheless, in tissue sections, an accurate measure of the fading of green fluorescence at the cellular level is difficult to detect due to the frequent superposition of adjacent cells and to the variability of EGFP expression between different cell types of the seminiferous epithelium [57]. To quantify the level of post-transcriptional silencing of EGFP in transfected cells, the reduction in green fluorescence from monocellular dispersions of seminiferous tubule cells from *in vivo *transfected testis was measured by fluorescence activated cell sorting (FACS). Red fluorescent cells were selected four days after transfection, and a reduction of 41.94% of green fluorescence was detected in the cells transfected with pGtoR compared to those transfected with pRed (used as a control). A significant difference in green fluorescence (t test; p = 0.047) was observed between red fluorescent cells depending on the vector used (pGtoR or pRed) (Fig. 3). This difference can only be interpreted as a specific silencing of EGFP mediated by shRNA-EGFP.

### Gene silencing in primary cultures of Sertoli cells

Since Sertoli cells were the main cell type transfected *in vivo*, transfection and gene silencing were assessed in cultured primary Sertoli cells. Sertoli cells from C57B/6J mice were independently transfected with pEGFP-N1, pGtoR or pRed vectors. Comparative analysis of the transfection efficiency showed that 38% of the cells had been transfected with pEGFP-N1 and 25% with pRed or pGtoR.

**Figure 4:**
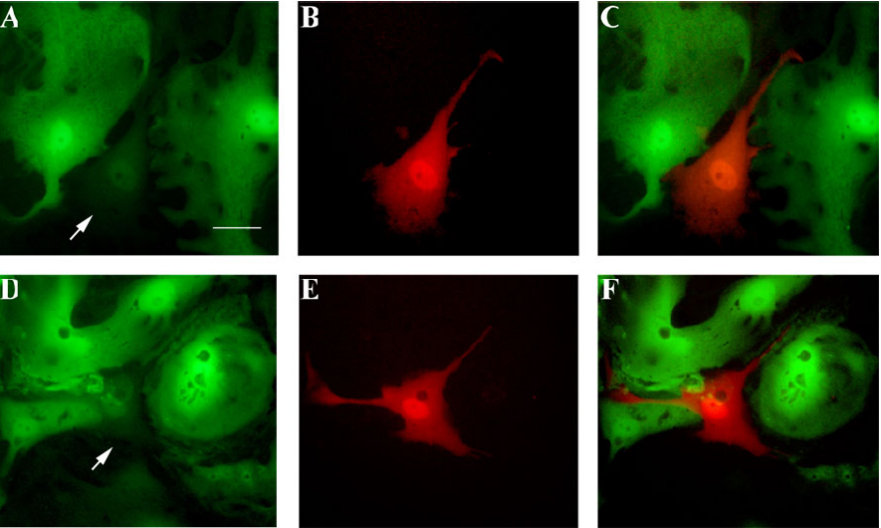
**Primary culture of Sertoli cells from EGFP transgenic mice (FM131) transfected with pGtoR**. The analysis was performed at 120 h (**A**, **B **and **C**) and 140 h (**D**, **E **and **F**) after transfection. Green fluorescence (excitation wavelength 488 nm) (**A **and **D**). Red fluorescence (**B **and **E**). Merge (**C **and **F**). Transfected cells (as demonstrated by red fluorescence) are indicated by arrows. Bar represents 10 μm.

In order to compare gene silencing by RNAi in cultured primary Sertoli cells and in *in vivo *transfected cells, Sertoli cells isolated from the EGFP transgenic mouse line FM131 were cultured. A reduction in green fluorescence due to EGFP protein was observed in the red fluorescent cells transfected with pGtoR compared to those that were not transfected (Fig. 4).

**Figure 5:**
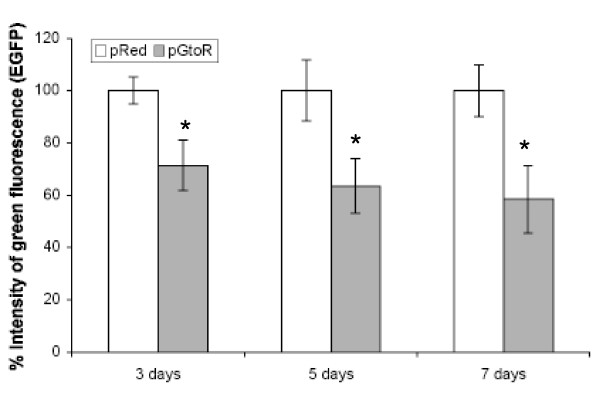
**Comparative results from flow cytometry analysis of EGFP-expressing cultured Sertoli cells *in vitro *transfected with the pGtoR or pRed**. The graph shows the mean (Mean ± SE) of green fluorescence intensity in cells transfected *in vitro *with the pRed or pGtoR, determined by flow cytometry at 3, 5 and 7 days after transfection. Asterisk shows statistically significant differences as measured by p < 0.05.

To indirectly quantify the silencing of EGFP, EGFP fluorescence intensity in transfected Sertoli cells was determined by using flow cytometry. Compared to the cells transfected with pRed, a significant reduction in EGFP fluorescence intensity was seen in the cells transfected with pGtoR at three (p = 0.0165), five (p = 0.0199) and seven days (p = 0.0171) post-transfection. The reduction of EGFP fluorescence was more significant at seven days (41.77%) and five days (36.55%) than at three days post-transfection (28.63%) (Fig. 5).

The efficiency of vector transfection *in vivo *after electroporation, the method employed here, was relatively low with all the vectors used. This might be attributed to an intrinsic characteristic of these cells or to the nature of the constructs used in these experiments. However, a preference for Sertoli cells and a similar transfection rate were observed with both the pEGFP-N1 and pGtoR vectors.

McCaffrey et al. [32] performed the first RNAi *in vivo *analyses in mammals. These authors used hydrodynamic injections to deliver siRNAs and shRNAs to the liver, but this method is limited to a number of highly vascularized tissues [31-39]. Other methods have been tested to deliver siRNAs to different organs, including lipid-based strategies [22-24,58] involving the use of siRNAs complexes with polyethyleneimine (PEI) [22], atelocollagen [59] and cholesterol [37]. Electroporation has also been used to efficiently deliver siRNA to the kidney [60], brain [61], eyes [62] and muscle [29].

In the mature mouse, Sertoli cells occupy approximately 15–20% of the volume of the seminiferous epithelium and a large proportion of the Sertoli cell surface is in contact with elongated spermatids and the tubular lumen [63]. If the access of seminiferous epithelium cells to transfecting molecules is via the tubular lumen, and the internalization of foreign DNA is mediated by the binding of DNA to the membrane [64], the Sertoli cells should be the most readily transfected cell type.

*In vivo *gene transfer to seminiferous epithelium cells has been conducted in the past using different strategies and with different purposes [51,65-74]. Yomogida et al. [51] used *in vivo *electroporation to introduce transgenes into Sertoli cells as a tool to investigate gene function during mammalian spermatogenesis. These authors microinjected the testis of 12 dpn (days post natal) mice because of the low number of differentiating germ cells in prepubertal animals, and obtained transfection of Sertoli cells and, to a small extent, of germ cells. In contrast, Shoji et al. [48] transfected tubular cells in mice aged 5–15 dpn mice, and found that most of the cells transfected were germ cells. Using similar experimental protocols, but in adult mice, we found a preferential transfection of Sertoli cells. Some of the different experimental conditions used in the *in vivo *testis transfection procedure might lay behind the differences in the proportion of cell types that were transfected. Furthermore, specific and complex structures define both the Sertoli-Sertoli and Sertoli-germ cell interactions within the mammalian seminiferous epithelium in adults, which cannot be controlled. The Sertoli-Sertoli and Sertoli-germ cells junctions may prevent other cell types from gaining access to the transfecting molecules. After Sertoli cells, elongated spermatids are the germ cells most likely to be transfected. However at this stage, elongated spermatids are in an advanced state of chromatin condensation and in the process of eliminating their cytoplasm, which reduces their volume by approximately 25%. This confers characteristics upon them that disables the entrance of transfecting molecules to the cytoplasm [75]. These dynamic interactions such as adhesion, attachment and communication between adjacent cells [42,76] explain the differences in the capacity of different cell types to be transfected *in vivo *during testis development.

Hasuwa et al. [50] developed a transgenic approach to deliver EGFP-targeted shRNAs into mice ubiquitously expressing EGFP. In this way, they studied the effectiveness of transgene-mediated gene silencing in different cells and tissues, however, no analysis in the testis was performed. We used the same vector and, as expected, the Sertoli cells were the main target cell type for transfection and EGFP silencing.

As the low efficiency of transfection of nonviral vectors is a technical limitation in the use of this approach to silence genes in seminiferous epithelium, alternative methodologies are also being explored.

## Conclusion

In conclusion, gene silencing by RNAi via shRNA, was demonstrated both *in vivo *and in primary culture of Sertoli cells. In Sertoli cells from the mouse model used, the reduction of 40% in the amount of target (EGFP) was significant. This also indicates that Sertoli cells have the necessary silencing machinery to repress the expression of endogenous genes via RNAi.

## Competing interests

The authors declare that they have no competing interests.

## Authors' contributions

EGG participated in designing the study and performed the experimental transfection of shRNAs. He also participated in the analysis and discussion of the results and drafted the manuscript. PPLC participated in the experimental design of the study, supervised the analysis, discussion of the results and critical revision of the manuscript. JdM was responsible for the conception, design, funding and supervision of this work. He also participated in the analysis and discussion of the results, drafting and critical revision of the manuscript. All authors read and approved the final manuscript.
